# Intranasal Delivery of Granisetron to the Brain via Nanostructured Cubosomes-Based In Situ Gel for Improved Management of Chemotherapy-Induced Emesis

**DOI:** 10.3390/pharmaceutics14071374

**Published:** 2022-06-29

**Authors:** Essam M. Eissa, Mohammed H. Elkomy, Hussein M. Eid, Adel A. Ali, Mohammed A. S. Abourehab, Amal M. Alsubaiyel, Ibrahim A. Naguib, Izzeddin Alsalahat, Amira H. Hassan

**Affiliations:** 1 Department of Pharmaceutics and Industrial Pharmacy, Faculty of Pharmacy, Beni-Suef University, Beni-Suef 62511, Egypt; essam.mohamed@pharm.bsu.edu.eg (E.M.E.); hussien.eid@pharm.bsu.edu.eg (H.M.E.); adel.ali@pharm.bsu.edu.eg (A.A.A.); amira.abdelatef@pharm.bsu.edu.eg (A.H.H.); 2 Department of Pharmaceutics, College of Pharmacy, Jouf University, Sakaka 72341, Saudi Arabia; 3 Department of Pharmaceutics, College of Pharmacy, Umm Al-Qura University, Makkah 21955, Saudi Arabia; maabourehab@uqu.edu.sa; 4 Department of Pharmaceutics and Industrial Pharmacy, Faculty of Pharmacy, Minia University, Minia 61519, Egypt; 5 Department of Pharmaceutics, College of Pharmacy, Qassim University, Buraidah 52571, Saudi Arabia; asbiel@qu.edu.sa; 6 Department of Pharmaceutical Chemistry, College of Pharmacy, Taif University, Taif 21944, Saudi Arabia; i.abdelaal@tu.edu.sa; 7 UK Dementia Research Institute Cardiff, School of Medicine, Cardiff University, Cardiff CF24 1TP, UK

**Keywords:** granisetron, cubosomes, in situ gel, biodistribution, brain targeting, pharmacokinetics, intranasal

## Abstract

This research aimed to boost granisetron (GS) delivery to the brain via the intranasal route to better manage chemotherapy-induced emesis. Glycerol monooleate (GMO), Poloxamer 407 (P 407) and Tween 80 (T 80) were used to formulate GS-loaded cubosomes (GS-CBS) utilizing a melt dispersion-emulsification technique. GS-CBS were characterized by testing particle diameter, surface charge and entrapment efficiency. The formulations were optimized using a Box–Behnken statistical design, and the optimum formula (including GMO with a concentration of 4.9%, P 407 with a concentration of 10%, and T 80 with a concentration of 1%) was investigated for morphology, release behavior, ex vivo permeation through the nasal mucosa, and physical stability. Moreover, the optimal formula was incorporated into a thermosensitive gel and subjected to histopathological and in vivo biodistribution experiments. It demonstrated sustained release characteristics, increased ex vivo permeability and improved physical stability. Moreover, the cubosomal in situ gel was safe and biocompatible when applied to the nasal mucosa. Furthermore, compared to a drug solution, the nose-to-brain pathway enhanced bioavailability and brain distribution. Finally, the cubosomal in situ gel may be a potential nanocarrier for GS delivery to the brain through nose-to-brain pathway.

## 1. Introduction

Emesis is a common and debilitating side effect of cytotoxic chemotherapy and radiation therapy [1,2]. As a result, conventional antiemetic medications are given in concomitance with chemotherapy and radiation, especially to patients who postpone or refuse these forms of therapy [3]. Following the discovery of 5-HT3 receptor antagonists (5-HT3 RAs), the treatment of emesis resulting from chemotherapy and radiation has vastly improved. This category of antiemetic agents has been recommended as a first-line treatment for emesis induced by chemotherapy and radiation [4].

Granisetron (GS) is a potent and selective 5-HT3 RA that efficiently relieves emesis arising from chemotherapy and radiation [5]. Additionally, it is used to prevent and alleviate postoperative emesis [6]. It inhibits the vagus nerve from activating the vomiting center in the medulla oblongata [7,8]. GS is a water-soluble drug having a 3–4 h half-life in healthy volunteers and a 9–12 h half-life in cancer patients [9]. Consequently, it is necessary to extend the half-life of GS in cancer patients. It is now available in several dosage forms, including intravenous injection, subcutaneous injection, oral tablet/solution and transdermal patch. For the oral and transdermal GS dosage forms, the onset of antiemetic effects of GS is relatively slow. Furthermore, individuals with impaired swallowing ability or nausea symptoms may find it difficult to take GS tablets by mouth. It also has a limited oral bioavailability because of hepatic first-pass metabolism [9]. GS injection is an invasive dosage form; thus, driving patients to face needless pain and the risk of infection due to injection, which is a serious concern for immunocompromised people. Therefore, it is essential to design an alternate rapid onset dosage form that can be delivered noninvasively for effective nausea and vomiting management.

In recent years, the nose-to-brain (NTB) route has been characterized as a noninvasive method for the fast transfer of drugs from the nasal cavity to the brain and bloodstream in order to treat CNS illnesses [10]. This is because the nose structure offers a large surface area with enough blood flow. The olfactory cells in the nasal mucosa also extend into the cerebral cavity [11]. When the delivery system touches the mucosa during nasal administration, the medicine is delivered directly to the brain, bypassing the blood brain barrier and into the systemic circulation, resulting in a rapid response [12,13]. Nevertheless, low instillation volume into the nasal cavity, poor absorption of hydrophilic molecules and rapid mucociliary washout are limitations of the intranasal route [14]. Previously, several studies investigated the potential of the nose-to-brain pathway in managing nausea and vomiting [15,16].

Liposomes, niosomes and cubosomes are examples of new nanovesicular systems that might be used to carry drugs from the nose to the brain. Their physical features make them a viable tool for shielding encapsulated drugs from degradation, boosting mucosal penetration, prolonging drug residency at the absorption site and managing drug release [17].

Cubosomes (CBS) are nanocarriers made up of a bicontinuous lipid bilayer separating two water channel networks. CBS are constituted of an amphiphilic polar lipid, like glycerol monooleate (GMO), which is the polar lipid often employed in creating CBS in the presence of a stabilizer [18]. Moreover, GMO-based lipids are nonirritant and nontoxic substances [14]. When the concentration of GMO in water surpasses the critical micelle concentration, it may self-assemble into a micellar structure and form a bicontinuous cubic structure [19,20]. CBS provides various benefits, including biocompatibility, high entrapment efficiency, thermodynamic stability, bioadhesive characteristics and controlled drug release [21]. In situ intranasal gel, which is a solution at 25 °C and a gel when delivered into the nose (32 to 34 °C), increases the nasal absorption and penetration rate by prolonging the nasal residency period of the formulation [22,23].

This research investigated the use of in situ nanostructured cubosomes to improve GS brain delivery through the nose-to-brain route. GS-CBS was developed, optimized using the Box–Behnken design, and assessed for different physical and morphological features to determine their suitability for the NTB route. The optimized formula was then incorporated into an in situ mucoadhesive gel to assure simple intranasal administration, increase residence duration, and improve absorption. Finally, the safety and in vivo biodistribution of the optimized formulation in situ gel and GS solution after intranasal delivery were examined.

## 2. Materials and Methods

### 2.1. Materials

Granisetron hydrochloride (GS) was received from SEDICO Pharmaceutical Company (Cairo, Egypt). Acetonitrile (HPLC grade), Poloxamer 407 (P 407), methanol (HPLC grade), glycerol monooleate (GMO) and diethyl ether were purchased from Sigma-Aldrich (St. Louis, MO, USA). Dialysis membrane (MW cut off: 12 kDa) was purchased from SERVA Electrophoresis GmbH (Heidelberg, Germany). Tween 80 (T 80) was obtained from El Gomhouria Co. (Cairo, Egypt). All other ingredients used were of analytical grade.

### 2.2. Methods

#### 2.2.1. Design and Optimization of Experiments

Utilizing Design Expert^®^ (Version 12.0.3.0, Stat-Ease Inc., Minneapolis, MN, USA), the impact of different formulation parameters on GS-CBS properties was investigated using a Box–Behnken (BB) design. GMO concentration (X_1_), which ranged from 3 to 7% (*w*/*v*) with respect to total dispersion weight, P 407 concentration (X_2_), which varied between 5 and 10% (*w*/*w*) from the dispersed phase, and T 80 concentration (X_3_), which varied between 1 and 3% (*w*/*w*) from the dispersed phase, were the independent variables. GS was used in an amount of 10 mg in all formulations. Particle size (PS), zeta potential (ZP), and entrapment efficiency (EE) were the dependent variables. As indicated in Table 1, the number of experimental runs was fifteen formulations (12 formulations and 3 repeated central points). Using the plot3D package in R software, 3D surface graphs are plotted [24,25]. The optimization was set to minimum size and maximum surface charge and entrapment to obtain the formula with the maximum desirability [26,27].

#### 2.2.2. Formulation of Granisetron-Loaded Cubosomes (GS-CBS)

Granisetron-loaded cubosomes (GS-CBS) were produced via the melt dispersion-emulsification strategy [28]. GMO was employed as the lipid phase, P 407 as the surfactant, and T 80 as the dispersion stabilizer beside P 407. The aqueous phase consisted of T 80 and GS (10 gm), which was warmed to 70 °C. GMO, as well as P 407 were melted in a water bath at 70 °C, gently injected into the warmed aqueous phase (70 °C) by an insulin syringe, and then emulsified for 10 min using an Ultra Turrax homogenizer (Ultra Turrax^®^ T 25 basic homogenizer, IKA, Staufen, Germany) at a speed of 8000 rpm. The resulting dispersion was kept at 25 °C for future study. Table 1 lists the components of the GS-CBS formulations according to BB.

#### 2.2.3. GS-CBS Characterization and Optimization

##### Particle Diameter and Zeta Potential Analysis

Utilizing a laser scattering approach (Zetasizer Nano ZS, Malvern Panalytical Ltd., Malvern, UK), the particle size (PS), polydispersity index (PDI), and zeta potential (ZP) of numerous GS-CBS formulations were evaluated [29]. Before analysis, the cubosomal suspensions were mixed with distilled water (1:10), and the evaluation was conducted at room temperature [30].

##### Measurement of Entrapment Efficiency

The unentrapped GS was separated from the cubosomal nanoparticles by centrifugation (15,000 rpm, 2 h, and 4 °C). The GS levels in the supernatant portion were estimated spectrometrically at 302 nm. The EE percentage was calculated using the following equation [26,27]:

$$\mathrm{EE}\%=\frac{\left(\mathrm{GS}\;\mathrm{added}\;\left(10\;\mathrm{mg}\right)-\mathrm{Free}\;\mathrm{GS}\;\mathrm{in}\;\mathrm{supernatant}\right)}{\mathrm{GS}\;\mathrm{added}\;\left(10\;\mathrm{mg}\right)}\times 100$$

#### 2.2.4. Characterization of the Optimized GS-CBS Formulation

##### Morphological Evaluation

The morphology of the optimum GS-CBS formula was evaluated via a transmission electron microscope (JEM-1400, Jeol, Tokyo, Japan) [26]. One drop of the optimized GS-CBS formula after dilution (1:10) was added onto a carbon-coated copper grid, left to dry, and then stained with phosphotungstic acid as a negative stain [31,32]. Finally, it was examined under TEM operating at 80 kV [33].

##### In Vitro Release of GS

GS release behavior was evaluated as previously published [23]. The bottom tip of the USP dissolution apparatus glass tubes (length: 7.5 and diameter: 2.5 cm) were sealed with a dialysis membrane. The glass tubes (donor chamber) were filled with various amounts of optimized GS-CBS and GS solution (GS-SOL) with similar concentrations of GS (3 mg). The tubes were then inserted into the USP dissolution apparatus I (Hanson, USA). Fifty mL of simulated nasal electrolyte solution (pH 5.5), made following the stated procedure [34], were added to the receptor chamber (maintained at 37 ± 0.5 °C and stirred at 50 rpm). One milliliter was collected from the receptor media at preset time intervals and replaced with an equal volume of fresh, simulated nasal electrolyte solution to maintain sink condition. The collected amount was filtered, and the GS released in the collected samples was measured using a spectrometer at 302 nm. The curve of percent of cumulative drug released vs. time was created.

##### Ex Vivo Permeation of GS

The permeation experiments were carried out using a Franz diffusion cell (2.5 cm^2^). The butcher supplied us with freshly removed nasal mucosa from sheep that had been carefully washed to eliminate any adherent tissues. After that, the mucosa was washed with PBS and soaked in it for 30 min [35]. A nasal mucosa of 0.2 mm thickness was positioned between the receptor and donor chambers. The donor chamber contained a precisely weighted volume (equivalent to 3 mg) of the optimal GS-CBS dispersion or GS-SOL. The receptor chamber contained 50 mL PBS (pH 6.5, maintained at 37 ± 0.5 °C, and stirred at 50 rpm). An amount of 1 mL from the receptor chamber was removed and replaced with an equal volume of fresh PBS. Furthermore, the samples were filtered and spectrometrically examined at 302 nm. Permeation parameters were calculated using a previously published method [30].

##### Short-Term Stability

The stability of the optimum GS-CBS formulation was tested by storing it for 90 days in the refrigerator. After formulation, a specified volume was collected from the optimized GS-CBS at defined time intervals. Particle diameter, zeta potential, and entrapment efficiency were assessed [30].

##### Preparation of GS-CBS Thermosensitive Gel

The optimum formula was integrated into a mucoadhesive gel formulation to provide convenient intranasal administration, increase residence duration, and hence improve absorption. The optimum formula and GS-SOL were incorporated into a mucoadhesive in situ gel base (Poloxamer 407 (20%), Poloxamer 188 (10%), and Carbopol 971P (0.5%)) using the cold technique [23].

#### 2.2.5. Evaluation of pH

The pH of the prepared GS-CBS in situ gel must be determined to guarantee that it does not irritate the nasal mucosa after intranasal application. The pH of the formulated GS-CBS in situ gel was estimated via the digital pH meter (Jenway, Cadmus Distribution Group Ltd., Chelmsford, UK).

#### 2.2.6. In Vivo Studies

Wistar male rats, weighing 215–255 gm, were used. All studies were performed with the agreement of the Local Institutional Animal Ethics Committee at Beni-Suef University (Approval No: 022-258) and followed the guidelines of the Declaration of Helsinki.

##### Histopathological Evaluation

Histological examination was performed to evaluate the safety of GS-CBS in situ gel after intranasal delivery and exclude nasal toxicity [36]. Six rats are divided into two groups. The first group received GS-CBS in situ gel (IN) twice daily for 7 days, while the other group served as a control [35]. The nasal mucosa of the sacrificed rats was meticulously detached after 7 days and submerged in formaldehyde (10%) for 24 h before being placed in paraffin blocks and cut into 5 mm thick sections. It was stained with hematoxylin and eosin (H&E) and inspected under the microscope [35].

##### In Vivo Biodistribution Analysis

Three groups of rats were assigned: group A received GS-SOL intravenously (IV), group B received GS-SOL in situ gel intranasally (IN), and group C received GS-CBS in situ gel intranasally (IN). After which, each group was divided into eight time-based subgroups (10, 15, 30, 45, 60, 120, 240, and 480 min). Each subgroup had three rats, each receiving a GS dose of 0.8 mg/kg. The rats were sedated by inhaling a sufficient amount of diethyl ether to avoid sneezing during intranasal instillation. For intravenous delivery, GS-SOL was injected into the tail veins of the animals. For intranasal studies, the dosage for GS-SOL in situ gel and GS-CBS in situ gel (0.80 mg/kg GS) was delivered by micropipette into both nostrils. After administration, the rats were sacrificed at different intervals by cervical dislocation, and blood was collected. Afterward, the brains were dissected, cleansed with 0.9% NaCl, and cleared of any adhering tissues or fluids [23].

##### Sample Preparation for Analysis

Plasma was isolated from the collected blood samples by centrifugation. The plasma was mixed with acetonitrile (1:1), resulting in the precipitation of proteins. The mixtures were vortexed (3 min) and then centrifuged for 25 min (11,000 rpm). The supernatant was then kept at −21 °C until analysis. In contrast, the brain was weighed, diluted with 0.9% NaCl (1:5), and homogenized. Acetonitrile (100–500 μL) was used to extract brain homogenates (1:1) which were vortexed (2 min) and then centrifuged for 25 min (4000 rpm and 4 °C). The supernatant was then kept at −21 °C until analysis.

##### Chromatographic Conditions

The quantitative analysis of GS was achieved by utilizing a validated LC-MS/MS method [37]. The analysis system consisted of an autosampler (SIL-20 AC), Shimadzu Prominence series LC system with degasser, and Zorbax C_18_ column (3.5 µm PS, 4.6 × 50 mm). The mobile phase was formic acid (0.1%) and acetonitrile (30:70). The autosampler and the column temperature were kept at 4 and 30 °C, respectively. The flow rate and the injection volume were 0.3 mL/min and 4 μL, respectively. The electrospray ionization source was set to operate in positive mode. Quantification was performed using the multiple reaction monitoring mode at a transition of *m*/*z* 313.1/138.1 Da with optimum collision energies of 22 eV. The assay demonstrated a linear calibration curve across the 1–20 ng/mL concentration range (R^2^ = 0.999).

##### Pharmacokinetic Analysis

GS pharmacokinetics following intranasal administration of the optimized GS-CBS and GS-SOL to rats were estimated using a noncompartmental model [38]. The pharmacokinetic parameters, including elimination rate constant (Ke), maximum concentration (C_max_), elimination half-life (t_1/2_), peak time (T_max_) and area under the curves (AUC), were computed by PK solver, an add-in program in Microsoft Excel. The drug targeting efficiency (DTE%) and drug transport percentage (DTP%) were used to calculate brain targeting efficiency. DTE% is the ratio of drug level in the brain acquired with IN administration vs. IV administration. DTP% is the proportion of the IN dose that is transported directly from the nose to the brain compared to the total quantity of drug reaching the brain following IN administration. Both DTE% and DTP% were computed as previously reported [23].

#### 2.2.7. Statistical Analysis

All research findings were shown as mean ± standard deviation. ANOVA was used to illustrate the significance of each factor, using the *aov* function in R software (version 4.2.0, R Core group, Bentley, Australia, 2022). The results were regarded as significant if *p*-value < 0.05.

## 3. Results and Discussion

### 3.1. Experimental Design and Optimization

**Figure 1:**
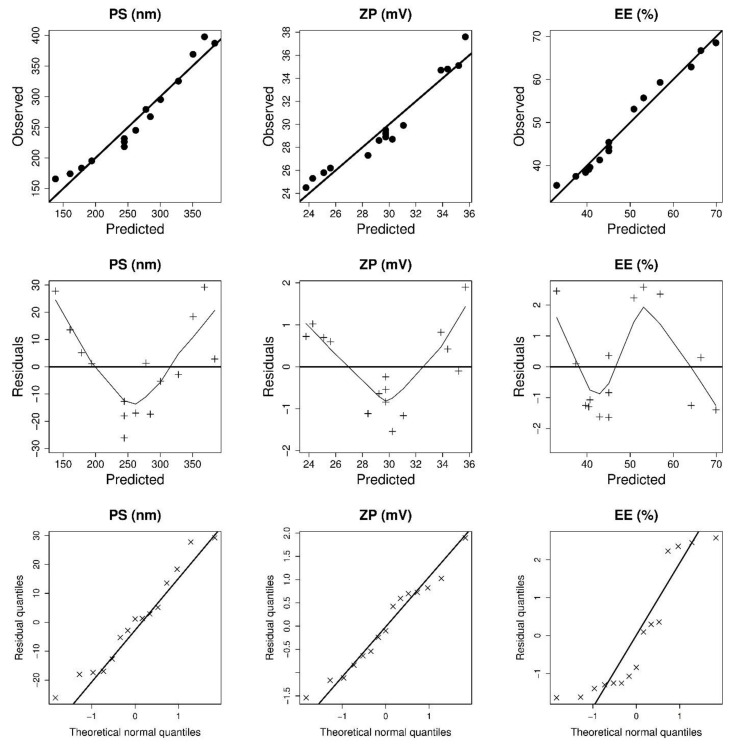
Model diagnostic plots of GS-CBS size (PS), surface charge (ZP), and entrapment efficiency (EE).

The results of PS, EE, and ZP of 15 experimental runs with various GMO, P 407 and T 80 concentrations are shown in Table 1. The wide range of dependent variables results implies that changes in GMO, P 407, and T 80 concentrations may considerably influence cubosomal features. Table 2 contains the mathematical equations in coded values that best characterize the relationships between the causal and the response factors. The negligible lack of fit shows that the models effectively explained the observed variance (Table 2). The Design-Expert program computed adequate precision to determine the reliability of the models used to navigate the design space [39]. Adequate precision in statistical analysis of all response variables was more than 4 (Table 2), and the predicted R^2^ results were close to the adjusted R^2^ results. Moreover, the ANOVA analysis indicates that the influence of GMO, P 407 and T 80 on the response variables was significant. Figure 1 shows the diagnostic model plots, which confirmed the adequacy of the fitted models.

#### 3.1.1. Analysis of Particle Size (PS)

**Figure 2:**
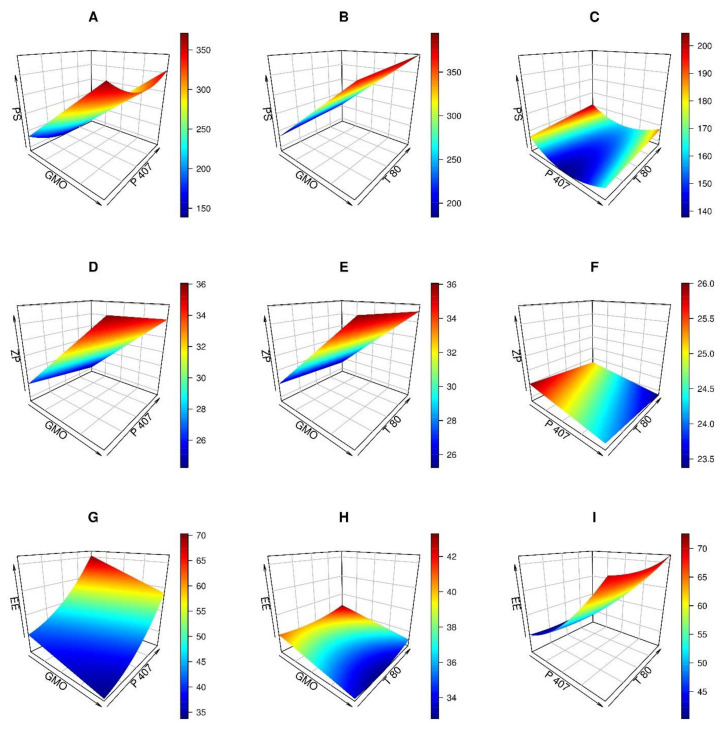
The 3D plots for the impacts of independent variables (glycerol monooleate (GMO), Poloxamer 407 (P 407), and Tween 80 (T 80)) on GS-CBS size (PS), surface charge (ZP), and entrapment efficiency (EE).

PS evaluation was performed to emphasize the nanoscale range of the produced cubosomal dispersions. As shown in Table 1, the PS of the produced cubosomal formulas varied between 165.8 and 397.6 nm. Using a reduced quadratic model, PS values were polynomially assessed. The influence of GMO and P 407 and T80 on the cubosomes’ PS is shown in Figure 2A–C. The PS increased as the level of GMO increased, as depicted in Figure 2A,B. Similar results were observed in previously published studies [40,41].

Furthermore, as shown in Figure 2A,C, increasing P 407 levels resulted in a substantial reduction in PS (*p*-value = 0.0267) until a particular level was reached. The lowest PS was observed when P 407 reached 7.7% *w*/*v*. The resulting reduction in size with increased P 407 levels might be attributed to reducing the interfacial tension between the lipid phase and the aqueous one, producing smaller particles [42]. Conversely, large particles were produced when the level of P 407 exceeded 10%, and it might be ascribed to the rupture of P 407/GMO ordering, hence reducing the steric stability of the dispersion [43]. Moreover, increasing the T 80 from 2 to 3% led to an increase in PS from 230 to 285 nm, Figure 2B,C. Increased viscosity of the dispersion may account for the considerable increase in PS at the highest T 80 level.

As previously noted, the PS greater than 100 nm was advised for brain targeting to suit the loading capacity of these nanocarriers over the BBB and minimize opsonization [44]. Nevertheless, it was believed that these cubosomes could pass the BBB safely and efficiently. The direct transfer of most nanocarriers from the nasal cavity to the brain may prevent or at least reduce opsonization, resulting in safe transport to the brain [45]. Furthermore, T 80 may help nanovesicles pass across the BBB [46]. Previous studies revealed that formulated nanoparticles with PS ranging from 100 to 300 nm were suitable for brain targeting [46].

#### 3.1.2. Analysis of Zeta Potential (ZP)

According to Table 1, the ZP values varied from −29.7 to −24.5 mV. The ZP might be seen as an indication of the physical stability of the dispersion. High surface charge values might protect nanoparticles against aggregation by imparting more repulsion forces to their surfaces [33]. Consequently, these results indicated that the formulated GS-CBS would provide a fair level of stability. The linear model was found to be suitable for analyzing the ZP data. ANOVA Type III-partial analysis revealed significant impacts of GMO and P 407 on ZP values (*p* < 0.05), but T 80 had no significant effect on ZP.

When the GMO level was raised from 3 to 7%, the ZP increased from −25 to −36 mV (Figure 2D,E). The ionization of the free oleic acid contained in GMO is responsible for the positive association between GMO concentrations and ZP values [47] as it increases the negative charge on the surface of the cubosomes. The rise in the P 407 level from 5 to 10% was followed by a modest decrease in ZP from −26 to −24 mV (Figure 2D,F).

#### 3.1.3. Analysis of Entrapment Efficiency (EE)

According to Table 1, the EE percentages for the developed cubosomal formulations varied from 35.4 to 68.5%. The reduced quadratic model was found to be appropriate for EE analysis. All the causal variables had a significant impact on EE. In addition, P 407 and T 80 positively affected EE from the regression coefficients, but GMO had a moderate effect.

Entrapment of more than 60% was obtained when the GMO level was at about 3% (Figure 2G,H), P 407 at more than 9% (Figure 2G,I), and T 80 at more than 1% (Figure 2H,I). The positive impact of P 407 and T 80 on EE may be explained by their capacity to coat nanocubosomes to stabilize them [46].

#### 3.1.4. Formulation Optimization

The Design expert^®^ software provided a range of suggestions that optimally satisfied the set constraints (minimum PS, maximum entrapment and surface charge). The selected formula (X_1_: GMO: 4.9% *w*/*v* %, X_2_: P 407: 10% *w*/*w* % and X_3_: T 80: 1% *w*/*w* %) had a desirability value of 0.59. Table 3 displays the values of experimental, predicted and prediction errors for the response variables of the optimized GS-CBS formula. Moreover, the computed prediction error percentage for all dependent variables was less than 10%. These findings demonstrated the validity of the final models.

**Figure 3:**
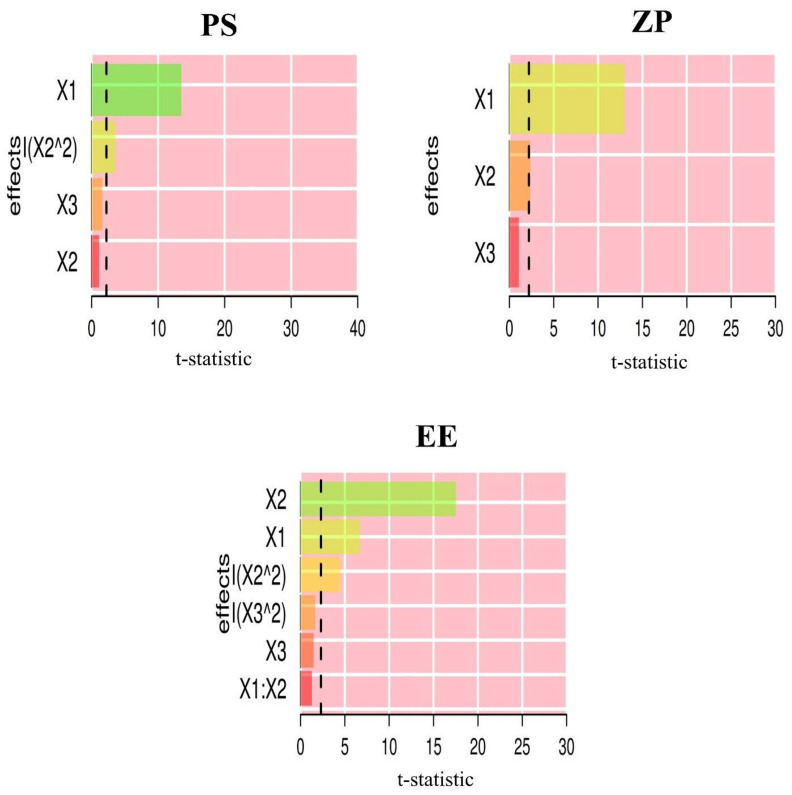
Pareto charts depicting the standardized impacts of independent variables: GMO concentration (X_1_), P 407 concentration (X_2_) and T 80 concentration (X_3_).

Pareto charts (Figure 3) show the standardized relative impacts of the formulation factors on PS, ZP and EE. GMO levels influenced PS and ZP more than P 407 and T 80 levels. However, the P 407 level was the most influential for EE.

### 3.2. Characterization of Optimized GS-CBS

#### 3.2.1. Morphological Evaluation

**Figure 4:**
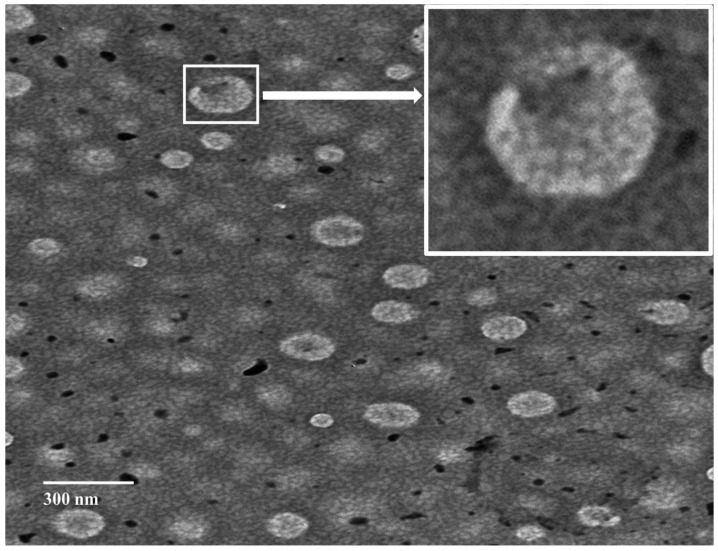
TEM morphology of the optimized formulation (GS-CBS).

The optimized GS-CBS formulation morphologically exhibited distinct cubic structures with no evidence of large aggregates (Figure 4), which may be attributed to the steric stability and electrostatic repulsion generated by the high ZP (−31.6). The average particle size was smaller when the TEM micrographs were compared to the Zetasizer results. This could be because TEM imaging was executed on dried materials [48].

#### 3.2.2. In Vitro Release of GS

**Figure 5:**
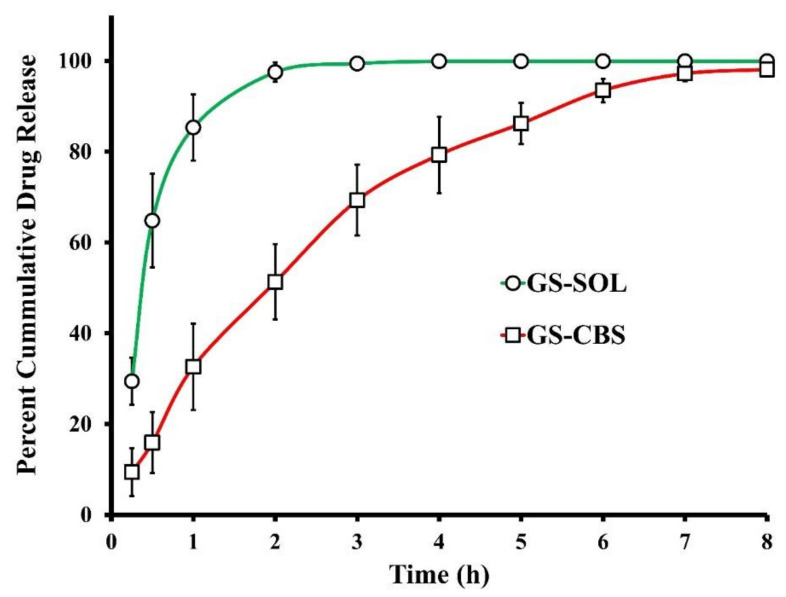
In vitro release profiles of GS from GS-CBS and GS-SOL.

The GS release patterns from the optimized GS-CBS formulation and BER-SOL were depicted in Figure 5. A total of 97.5% of the drug was released by BER-SOL after 2 h, but the optimized GS-CBS released only 51.3% within the same time. The release behavior of the optimized GS-CBS formula displayed a small initial burst followed by an extended period during the experiment duration (8 h). This initial burst may be explained by the release of GS existing below or at the cubosomal surface [42] and by the hydrophilic coating imparted by T 80 [49]. In contrast, the subsequent extended release resulted from the GS being entrapped inside the peculiar cubosomal structure. Furthermore, since GMO is a major component of cubosomes, GS may have partitioned slowly from the oily matrix to the aqueous matrix. In contrast, GS diffused rapidly from the solution to the receptor medium in the GS-SOL.

#### 3.2.3. Ex Vivo Permeation of GS

**Figure 6:**
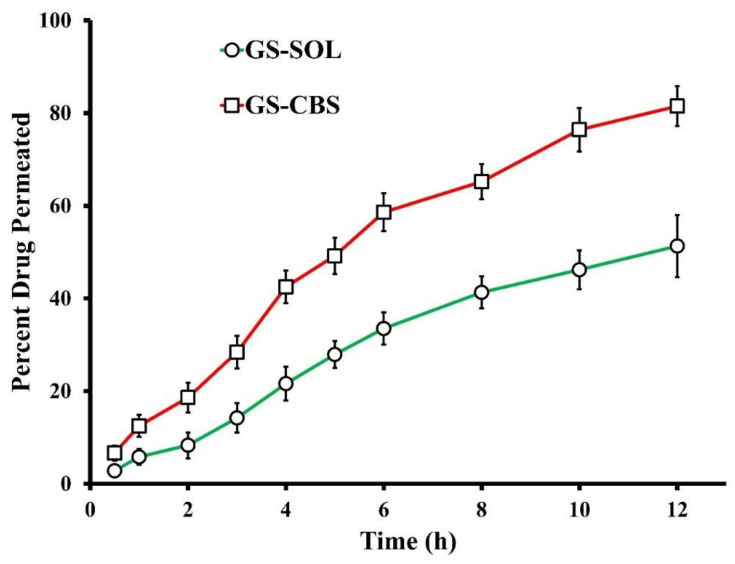
Ex vivo permeation profiles of GS from GS-CBS and GS-SOL.

GS permeation characteristics from the optimized GS-CBS and GS-SOL formulations are shown in Figure 6. The capacity of the optimized GS-CBS formulation and GS-SOL to penetrate the contained drug via the nasal membrane of sheep were compared. As demonstrated in Figure 6, drug permeation from the optimized GS-CBS formulation was much greater in both rate and extent than the GS-SOL. These outcomes may be attributed to the nanoscale and the presence of T 80, which acts as a permeation enhancer [50]. Moreover, T 80 may make the cubosomal wall elastic, enabling nanovesicles to enter through tiny pores in the nasal membrane [46]. In addition, the flux (Jss) was estimated at the end of the test for comparative reasons. There was a significant difference in Jss between the optimized GS-CBS (45.9 µg cm^−2^ h^−1^) and GS-SOL (27.1 µg cm^−2^ h^−1^), as disclosed in Table 4. This might also be reflected by the estimated permeability coefficient, which was about two times greater for the optimized GS-CBS than for GS-SOL, for drug penetration through the nasal membrane (Table 4).

#### 3.2.4. Short-Term Stability

**Figure 7:**
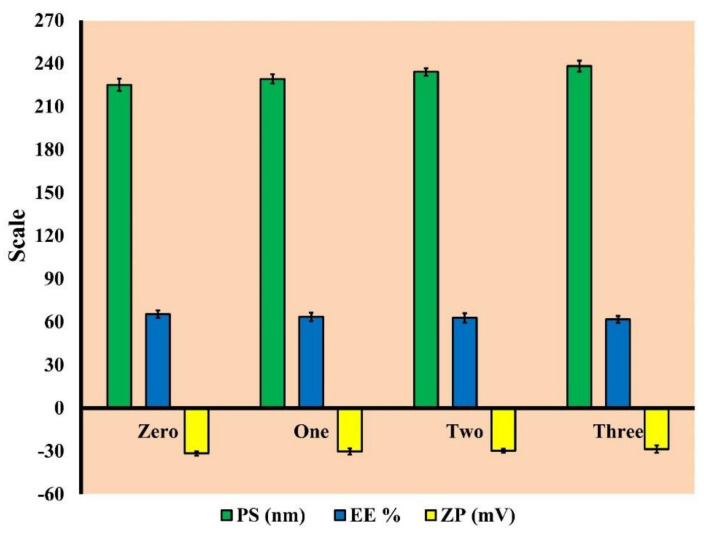
The PS, EE and ZP of the optimized formulation (GS-CBS) after three months of storage.

The milky white appearance of the stored optimized GS-CBS formulation was maintained throughout the storage duration (90 days), with no phase separation or aggregate development. Figure 7 depicts PS, EE, and ZP changes throughout the storage time of the optimized GS-CBS formulation. There was a slight decline in EE and ZP, and an increase in PS, which was deemed negligible (*p* > 0.05) as per our statistical analysis. These findings may be ascribed to the complex arrangement of two synergistic stabilizers (P 407 and T 80). In addition, the lipid inside the matrix of CBS preserved the cubic structure shape [51]. The high ZP of the optimized formulation provided further stability (31.6 ± 0.2 mV).

#### 3.2.5. Evaluation of pH

Estimating the pH of the optimized GS-CBS is essential to verify that it is safe on intranasal mucosa. The normal nasal mucosa pH is ranged between 4.5 and 6.5, whereas the pH of the optimized GS-CBS was reported to be 6.1. These findings indicate that the GS-CBS formulation is suitable for intranasal delivery.

#### 3.2.6. Nasal Histopathological Studies

**Figure 8:**
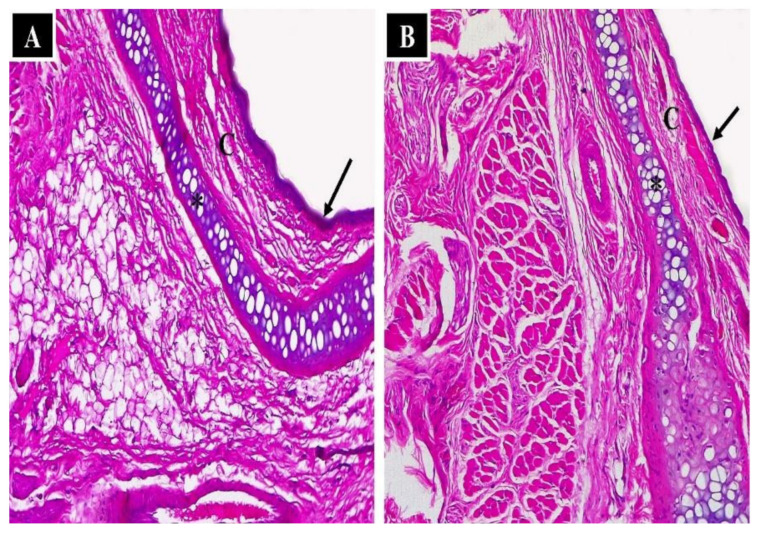
Light photomicrographs show (**A**) nasal epithelium of the control group (without any treatment) and (**B**) nasal epithelium of the GS-CBS in situ gel group. Notice normal nasal cartilage (*), normal nasal epithelium (arrows) and capillaries (C). H&E stain ×200.

A histopathological assessment was conducted on the mucosal membrane to determine the safety of the applied formulations. A negative control was employed to compare and determine whether the administered formulation irritated the membrane. Figure 8A shows the mucosal structure of the negative control, which included normal nasal epithelium (arrows), capillaries (C) and normal nasal cartilage. Similarly, normal histological assembly (normal nasal epithelium, capillaries, and normal nasal cartilage) was detected in the nasal mucosa of the rats following administration of the optimized GS-CBS formula, as shown in Figure 8B. This might be attributable to the biocompatible ingredients in the cubosomal composition.

#### 3.2.7. In Vivo Biodistribution Analysis

**Figure 9:**
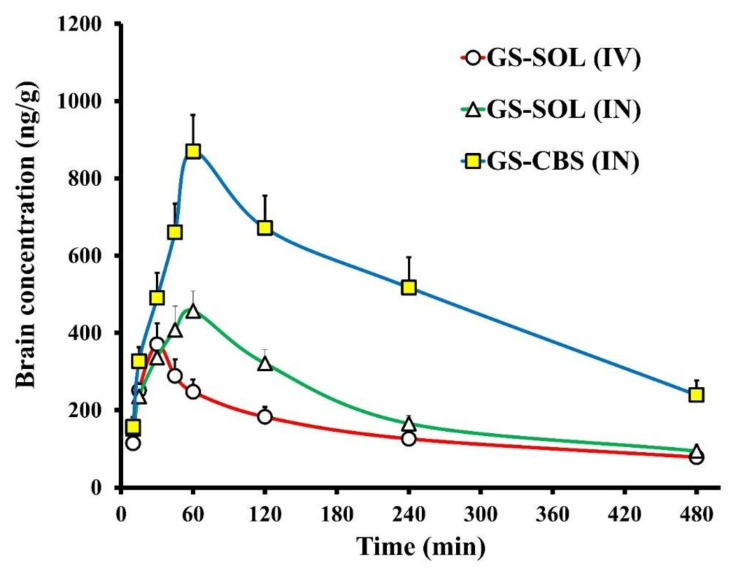
Granisetron levels in rat brain following GS-SOL (IV), GS-SOL (IN) and GS-CBS (IN) administration.

**Figure 10:**
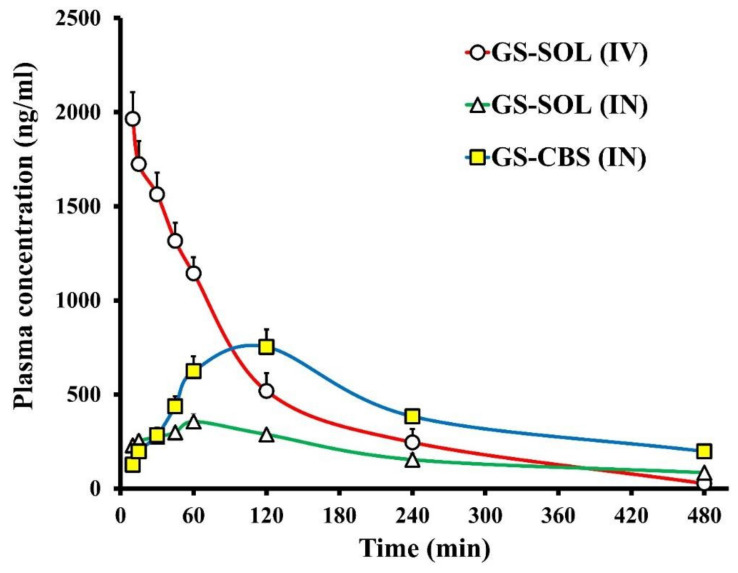
Granisetron levels in rat plasma following GS-SOL (IV), GS-SOL (IN) and GS-CBS (IN) administration.

The pharmacokinetics of GS were investigated in the brain and plasma after intranasal administration of GS-CBS in situ gel and GS-SOL in situ gel and intravenous administration of GS-SOL. Figure 9 and Figure 10 depict the in vivo drug behavior in the brain and plasma, respectively. Table 5 shows the pharmacokinetic parameters of GS-CBS in situ gel (IN), GS-SOL in situ gel (IN) and GS-SOL (IV). The pharmacokinetic data revealed that the GS level in the plasma reached its peak after 60 and 120 min consequent to the administration of GS-SOL in situ gel (IN) and GS-CBS in situ gel (IN), respectively. Since intranasal administration leads to systemic drug absorption, the presence of GS in plasma is expected [52]. In contrast, after 10 min of intravenous administration, the plasma concentration reached its peak and then declined rapidly over the next 2 h. The level of GS in the brain reached its peak after 30, 60, and 60 min upon administration of GS-SOL (IV), GS-SOL in situ gel (IN) and GS-CBS in situ gel (IN), respectively. The plasma C_max_ of GS-SOL (IV) was 1964 ± 142 ng/mL, GS-SOL in situ gel was 356 ± 41 ng/mL and GS-CBS in situ gel was 752 ± 93 ng/mL. The brain C_max_ of GS-SOL (IV) was 370.8 ± 54 ng/mL, GS-SOL in situ gel was 457.2 ± 52 ng/mL and GS-CBS in situ gel was 869.4 ± 95 ng/mL.

C_max_ was greater in brain and plasma for GS-CBS in situ gel (IN) compared to GS-SOL in situ gel (IN). Furthermore, the GS bioavailability was significantly improved by GS-CBS in situ gel, as seen by the increased AUC_0–480_ values (Table 5). GS-CBS in situ gel revealed a relative bioavailability of 221% in plasma and 248% in the brain compared to GS-SOL in situ gel. This may be related to the sustained release and the high residence time of GS-CBS in situ gel [53,54], resulting in low initial concentration but subsequently greater bioavailability. Furthermore, the GS-CBS in situ gel revealed much better bioavailability (299%) in the brain than the GS-SOL (IV). These findings may be explained by the direct olfactory transport of the nasal formulation to the brain, bypassing the BBB.

As demonstrated in Table 5, brain/blood ratios were computed for concentration and AUC_0-480_ for assessment of brain targeting and comparative purposes. The GS-CBS in situ gel (IN) showed higher ratios when compared to GS-SOL (IV) and GS-SOL (IN). This might be because oily GMOs were included, which enhanced lipophilicity and propensity for BBB permeability [55]. In addition, the presence of T 80 may increase the flexibility and binding efficiency to apolipoprotein E, enabling cubosomes to collapse and pass through pores of smaller size, thus increasing BBB permeability [46,56,57].

The GS-CBS in situ gel had a much longer elimination half-life compared to the GS-SOL in situ gel. This might be explained by the hydrophilic coating provided by T 80. This coating might prevent the opsonization of various antibodies, and so macrophage engulfment [58]. Therefore, T 80 may enhance particle circulation and medication residence-time inside the body.

The DTP and DTE are used to assess the amount of drug entering the brain via the olfactory pathway. These were calculated based on the distribution to the tissue/organs after IV and IN administration. The GS-CBS in situ gel had the greatest DTE and DTP, at 270 and 63%, respectively, suggesting that the cubosomal formulations improved GS brain targeting over GS-SOL in situ gel.

## 4. Conclusions

The optimized cubosomal dispersion with minimum size and maximum surface charge and entrapment was transformed into an in situ gel in order to boost the residence duration on the nasal mucosa and enhance physical stability. The histopathological analysis revealed that the optimized cubosomal in situ gel is safe and tolerable. Furthermore, compared to a drug solution, it produced significantly improved GS penetration across the nasal membrane, greater bioavailability and better brain distribution following intranasal delivery. Based on these results, cubosomal in situ gel may be a suitable nanocarrier for intranasal brain targeting of GS for improved control of chemotherapy-induced emesis.

## Figures and Tables

**Table 1:** Independent variables, experimental runs, and response variables for the formulations of GS-CBS according to the Box–Behnken design.

**Independent Variables**		**Levels**					
		**(−1)**		**(0)**		**(1)**	
X_1_: GMO (*w*/*v* %) ^a^		3		5		7	
X_2_: P 407 (*w*/*w* %) ^b^		5		7.5		10	
X_3_:T 80 (*w*/*w* %) ^b^		1		2		3	
**Run**	**GMO** **(*w*/*v* %)**	**P 407** **(*w*/*w* %)**	**T 80** **(*w*/*w* %)**	**Y_1_: Particle Size (nm)**	**Y_2_: Entrapment Efficiency (%)**	**Y_3_: Zeta Potential (mV)**	**Y_4_: Polydispersity Index ^c^**
1	0	0	0	218.3 ± 2.4	43.4 ± 2.7	(−) 29.5 ± 1.4	0.452 ± 0.13
2	0	1	1	267.3 ± 3.1	66.7 ± 3.2	(−) 27.3 ± 2.1	0.051 ± 0.03
3	0	0	0	226.4 ± 2.6	44.2 ± 2.8	(−) 28.9 ± 1.5	0.472 ± 0.15
4	−1	1	0	183.5 ± 1.9	68.5 ± 3.1	(−) 24.5 ± 1.3	0.437 ± 0.21
5	1	0	1	369.1 ± 3.7	41.3 ± 2.5	(−) 34.8 ± 2.3	0.494 ± 0.17
6	0	1	−1	245.1 ± 2.7	62.9 ± 2.4	(−) 28.6 ± 2.5	0.274 ± 0.09
7	−1	−1	0	195.3 ± 2.3	39.1 ± 1.8	(−) 26.2 ± 1.3	0.428 ± 0.22
8	0	−1	1	295.2 ± 4.2	38.4 ± 2.1	(−) 28.7 ± 2.4	0.476 ± 0.16
9	−1	0	−1	165.8 ± 1.6	53.1 ± 2.7	(−) 25.8 ± 1.2	0.433 ± 0.24
10	1	0	−1	325.3 ± 2.9	39.6 ± 2.3	(−) 35.1 ± 3.1	0.198 ± 0.08
11	0	−1	−1	279.2 ± 3.1	37.5 ± 1.8	(−) 29.9 ± 2.6	0.518 ± 0.25
12	1	−1	0	387.1 ± 3.9	35.4 ± 2.6	(−) 37.6 ± 3.2	0.854 ± 0.31
13	1	1	0	397.6 ± 2.5	59.3 ± 2.9	(−) 34.7 ± 2.1	0.071 ± 0.04
14	0	0	0	231.7 ± 3.8	45.4 ± 2.4	(−) 29.2 ± 3.3	0.194 ± 0.11
15	−1	0	1	174.2 ± 2.4	55.7 ± 3.1	(−) 25.3 ± 1.7	0.492 ± 0.21

GMO: Glyceryl monooleate; P 407: Poloxamer 407; T 80: Tween 80. ^a^ with respect to the total dispersion weight. ^b^ with respect to the concentration of the dispersed phase. ^c^ not included in optimization.

**Table 2:** The outcomes of all statistical analyses of response variables.

Source	PS		ZP		EE	
	F-Value	*p*-Value	F-Value	*p*-Value	F	*p*-Value
Model	205.09	<0.0001	152.80	<0.0001	104.05	<0.0001
X_1_: GMO (*w*/*v* %)	746.25	<0.0001	439.97	<0.0001	64.53	<0.0001
X_2_: P 407 (*w*/*w* %)	6.73	0.0267	14.77	0.0027	473.33	<0.0001
X_3_: T 80 (*w*/*w* %)	8.50	0.0154	3.67	0.0818	4.41	0.0689
X_1_ X_2_					12.10	0.0083
X_2_^2^	58.87	<0.0001			67.71	<0.0001
X_3_^2^					4.29	0.0720
Lack of Fit	1.45	0.4703	5.36	0.1670	7.02	0.1298
Model	Reduced Quadratic		Linear		Reduced Quadratic	
Adjusted R^2^	0.9831		0.9702		0.9779	
R^2^	0.9880		0.9766		0.9873	
%CV	1.85		2.20		10.48	
Predicted R^2^	0.9730		0.9508		0.9416	
Adequate precision	43.7095		33.9851		30.8347	
Standard deviation	0.0012		0.0008		14,008.91	
$1/\mathrm{Sqrt}\;\left(\mathrm{PS}\right)=0.066-0.011\cdot X_{1}+0.0012\cdot X_{2}-0.0013\cdot X_{3}-0.005\cdot X_{2}^{2}$						
$1/\left(\mathrm{ZP}\right)=0.034-0.006\cdot X_{1}+0.001\cdot X_{2}+0.0005\cdot X_{3}$						
${\left(EE\right)}^{3}=93,786.5-39,786.5\cdot X_{1}+107,756\cdot X_{2}+10,400.6\cdot X_{3}-24,369.2\cdot X_{1}\cdot X_{2}+59,813.4\cdot X_{2}^{2}+15,60.6\cdot X_{3}^{2}$						

**Table 3:** The values of experimental, predicted and prediction error of the optimized GS-CBS formulation dependent variables.

	Vesicle Size (nm)	Zeta Potential (mV)	Entrapment Efficiency%
Experimental value	225.2	(−) 31.6	65.4
Predicted value	242.8	(−) 28.5	63.6
Prediction error (%) ^£^	7.82	9.81	2.75

^£^ Computed as (Experimental-Predicted)/Experimental × 100.

**Table 4:** Ex vivo permeation parameters of GS-SOL and GS-CBS.

Formulation	Cumulative GS Permeated at 12 h (μg/cm^2^)	Permeability Coefficient (cm/h)	Flux (Jss) (µg cm^−2^ h^−1^)
GS-SOL	615.6 ± 41.3	0.02704 ± 0.00013	27.1 ± 1.67
GS-CBS	978.4 ± 51.9	0.04589 ± 0.00043	45.9 ± 3.84

**Table 5:** Pharmacokinetic parameters of GS-CBS in situ gel (IN), GS-SOL in situ gel (IN), and GS-SOL (IV).

Formulation	Tissue/Organ	C_max_ (ng/mL)	T_max_ (min)	t_1/2_ (min)	Ke (min^−1^)	AUC_0–t_ (ng/mL·min)	AUC_brain_/AUC_blood_	C_brain_/C_blood_ at 30 min
GS-SOL (IV)	Brain	370.8	30	301	0.0023	105,670	0.46	0.237
	Blood	1964	10	77	0.0090	227,502		
GS-SOL in situ gel (IN)	Brain	457.2	60	188	0.0037	127,598	1.12	1.228
	Blood	356	60	200	0.0035	113,892		
GS-CBS in situ gel (IN)	Brain	869.4	60	231	0.0030	316,669	1.26	1.733
	Blood	752	120	193	0.0036	252,287		

## Data Availability

Data sharing contains in this article.
